# A Comparison of Central Composite Design and Taguchi Method for Optimizing Fenton Process

**DOI:** 10.1155/2014/869120

**Published:** 2014-08-27

**Authors:** Anam Asghar, Abdul Aziz Abdul Raman, Wan Mohd Ashri Wan Daud

**Affiliations:** Chemical Engineering Department, Faculty of Engineering, University of Malaya, 50603 Kuala Lumpur, Malaysia

## Abstract

In the present study, a comparison of central composite design (CCD) and Taguchi method was established for Fenton oxidation. [Dye]_ini_, Dye : Fe^+2^, H_2_O_2_ : Fe^+2^, and pH were identified control variables while COD and decolorization efficiency were selected responses. *L*_9_ orthogonal array and face-centered CCD were used for the experimental design. Maximum 99% decolorization and 80% COD removal efficiency were obtained under optimum conditions. *R* squared values of 0.97 and 0.95 for CCD and Taguchi method, respectively, indicate that both models are statistically significant and are in well agreement with each other. Furthermore, Prob > *F* less than 0.0500 and ANOVA results indicate the good fitting of selected model with experimental results. Nevertheless, possibility of ranking of input variables in terms of percent contribution to the response value has made Taguchi method a suitable approach for scrutinizing the operating parameters. For present case, pH with percent contribution of 87.62% and 66.2% was ranked as the most contributing and significant factor. This finding of Taguchi method was also verified by 3D contour plots of CCD. Therefore, from this comparative study, it is concluded that Taguchi method with 9 experimental runs and simple interaction plots is a suitable alternative to CCD for several chemical engineering applications.

## 1. Introduction

Fenton oxidation is an efficient and widely applied AOPs, which utilizes ferrous iron (Fe^+2^) and hydrogen peroxide (H_2_O_2_) under acidic conditions to produce hydroxyl radical (HO^•^) (reaction 1). Formation of HO^•^ is advantageous because it is highly oxidative (*E* = 2.8 eV) and nonselective in nature [1, 2]. However, the performance of Fenton reaction is influenced by several parameters such as pH, reaction time, initial concentrations of Dye, H_2_O_2_, and Fe^+2^ catalysts [3, 4]. Moreover, excess consumption of chemicals (Fe^+2^ salt, H_2_O_2_ acid/base) and high cost of H_2_O_2_ have made this process economically nonviable [5, 6]. Therefore, a systematic way of planning, execution, and statistical evaluation of process is required which is only possible through optimization process [7]. Consider

(1) $$\begin{array}{c} {\text{Fe}}^{+2}+{\text{H}}_{2}{\text{O}}_{2}⟶{\text{Fe}}^{+3}+{\text{HO}}^{•}+{\text{OH}}^{-} \end{array}$$

**Figure 1:**
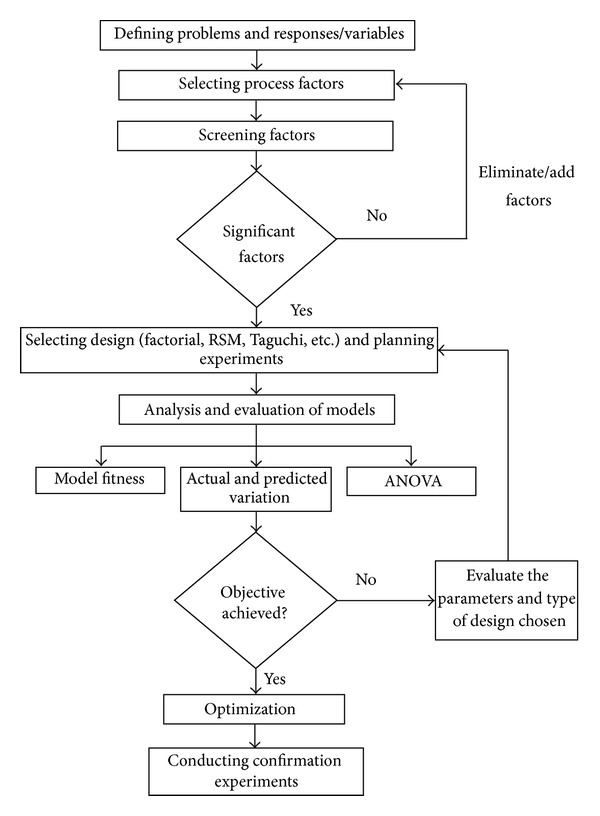
Flow chart for optimization.

In context to any engineering problem, optimization refers to improving the performance of system or process by applying several variables in different combinations to get the best possible result [8]. Optimization techniques are broadly classified into two categories: univariate and multivariate approach. Univariate approach, also known as one-factor-at-time (OFAT) approach, involves variation of one parameter at a time. Nevertheless, multivariate approach has several advantages over OFAT. For example [9], (1) it provides global knowledge in its whole experimental domain while OFAT gives local knowledge where experiment is performed; (2) it is possible to study the interaction between the factors and nonlinear relationships with the responses, (3) the number of experiments required to optimize the process is considerably lesser than that of OFAT approach; (4) within the experimental domain at each point, quality of the information is higher and can be known through the leverage. The stepwise execution of optimization is presented in Figure 1.

Driven by the need of reducing the number of experiments, cost, time, and physical efforts, design of experiment (DOE) is an important statistical and mathematical tool for solving complex and multifactor engineering problems. It includes response surface methodology (RSM), factorial design, and in some special cases artificial neural network (ANN) [10]. However, central composite design (CCD), D-optimal, and Box-Behnken are found to be widely used optimization techniques for Fenton oxidation [11–13] because of the advantage of optimizing multifactor problems with optimum number of experimental runs. Besides, these methods have limitation of increased number of experiments if several factors were selected for process optimization. For example, Box-Behnken suggests 54 experiments if six factors are required to be studied and with CCD this number increases up to 80. Thus, with multiple factors these techniques are not appropriate because it increases the cost of chemicals, time, and physical efforts. Therefore, a simplified design strategy is required that can be used to overcome these problems.

Taguchi method is a robust statistical tool that allows the independent evaluation of the responses with minimum number of experiments. It employs orthogonal arrays for experimental design and *S*/*N* ratio instead of responses itself to determine the optimum settings of control factors and thus neglects the variations caused by uncontrollable factors [14]. With this method, experimental results can be analyzed through *S*/*N* ratio and ANOVA with simultaneously evaluating the significance of the factors in terms of their contribution to the response values.

Available works on optimization of Fenton oxidation mainly focus on CCD under RSM [15–17]. Nevertheless, fewer studies based on Taguchi method are found in the literature [18–20] but which technique is better for Fenton oxidation is still not inclusive. Therefore, this study was planned to compare two experimental design techniques and Fenton oxidation with four parameters such as [pollutants]_ini_, Dye : Fe^+2^, H_2_O_2_ : Fe^+2^, and pH was selected as a case study. For comparison, frequently used CCD and robust Taguchi experimental design technique were chosen with a special focus on comparing the optimization and detailed statistical analysis of the experimental results.

## 2. Background

### 2.1. Response Surface Methodology and Central Composite Design

RSM, a multivariate statistical tool, consists of a group of mathematical and statistical techniques that are based on the fit of empirical models to the experimental data obtained in relation to experimental design [21]. It employs lower order polynomial [22] and it has already been proved to be a reliable statistical method for chemical process applications [23, 24].

In RSM category, CCD which is appropriate for fitting second order polynomial equations has been frequently discussed for optimizing several research problems. A CCD has three groups of design points [25]:

- two-level factorial or fractional factorial design points (2^*k*^), consisting of possible combinations of +1 and −1 levels of factor;
- 2*k* axial points (sometimes called star points) fixed axially at a distance say *α* from the center to generate quadratic terms;
- center points which represent replicate terms; center points provide a good and independent estimate of the experimental error.

**Figure 2:**
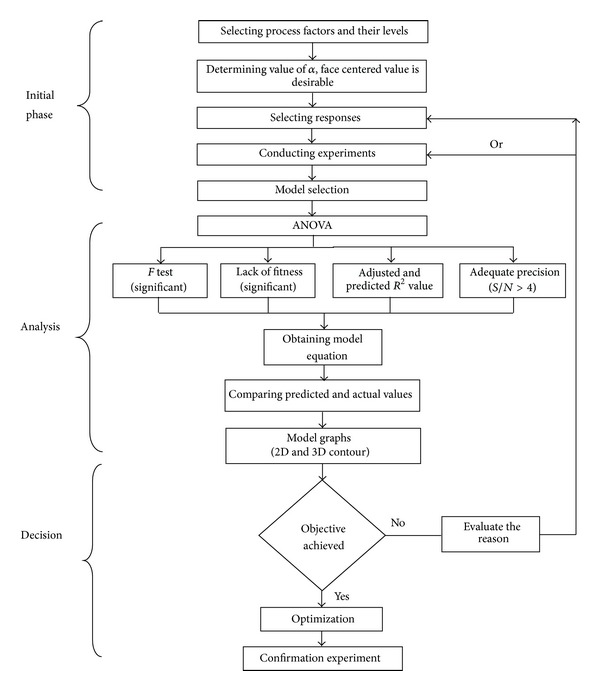
Central composite design flow diagram.

Considering these points, the number of experiments designed by CCD will be

(2) $$\begin{array}{c} N=k^{2}+2k+n, \end{array}$$

where *N* is the total number of experiments, *k* is the number of factors studied, and *n* is the number of replicates. Central composite design under RSM is normally performed either by using design expert software or Minitab. In this study, design expert (Version: 8.0.7.1) is used as optimization software. The steps that will be followed for the central composite design (CCD) are presented in Figure 2.

In CCD, value of alpha is important to calculate as it could determine the location of axial points in experimental domain. Depending on alpha value, design is spherical, orthogonal, rotatable, or face centered. Practically, it is in between face centered and spherical and is calculated as

(3) $$\begin{array}{c} \alpha ={\left(2^{k}\right)}^{0.25}. \end{array}$$

Value of alpha equals 1 is desirable because it ensures the position of axial point within factorial portion region. It is called face centered design and offers three levels for the factors to be put in the experimental design matrix.

Experimental results obtained are analyzed using response surface regression procedure of statistical analysis system. Corelation between responses and independent variables is obtained by fitting them into second order polynomial equation [10]

(4) $$\begin{array}{c} Y=\beta_{0}+\overset{k}{\underset{i=1}{\sum }}\beta_{i}x_{i}+\overset{k}{\underset{i=1}{\sum }}\beta_{ii}x_{ii}^{2}+\overset{k}{\underset{i=1}{\sum }}\overset{k}{\underset{i\neq j=1}{\sum }}\beta_{ij}x_{i}x_{j}+\varepsilon . \end{array}$$

Here, *Y* represents the responses, *k* is the total number independent factors, *β*_0_ is an intercept, *i*, *ii*, and *ij* with *β* represent the coefficient values for linear, quadratic, and interaction effects, respectively, and *x*_*i*_ and *x*_*j*_ in the above equation show the coded levels for independent variables [26].

### 2.2. Taguchi Method and Signal to Noise Ratio

**Figure 3:**
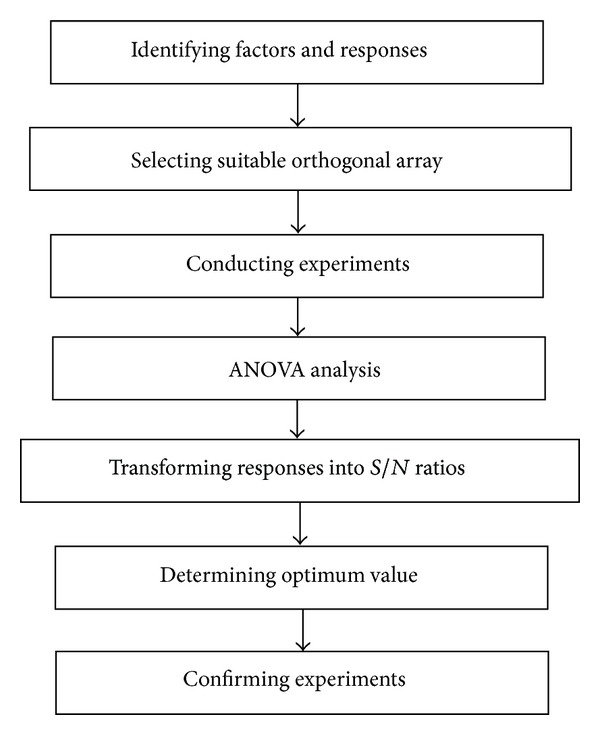
Taguchi orthogonal array.

Taguchi method is a robust and multiparameter optimization statistical technique which employs fewer numbers of experiments to identify and optimize parameters to achieve desired response [27, 28]. It is a simple and systematic way of determining the effect of factors on responses and optimum condition of the factors. Taguchi method utilizes full fractional design called orthogonal arrays and ANOVA as a tool for analysis [29]. Orthogonal arrays are the minimum set of experiments which represents the various combinations of factors. Output of the orthogonal arrays is optimized with respect to signal to noise ratio (*S*/*N*) of the responses instead of the responses itself and thus it reduces the process variability [30]. This feature marks the difference between the conventional statistical technique and Taguchi method. The stepwise procedure for Taguchi method is represented in Figure 3.

In Taguchi method, *S*/*N* ratio is the measure of the deviation of the response from the desired value. Here, “signal” implies the mean value while “noise” shows the standard deviation term. It means that lower variability in the process is ensured through maximizing the *S*/*N* ratio. However, depending on the type of response desired, Taguchi classified *S*/*N* ratio into three categories: smaller-the-better, larger-the-better, and nominal-the-better [31]:

 Smaller is better:
  (5) $$\begin{array}{c} S/N=-10\log\left(\frac{1}{n}\overset{n}{\underset{k=1}{\sum }}y_{i}^{2}\right). \end{array}$$
 Larger is better:
  (7) $$\begin{array}{c} S/N=-10\log\left(\frac{1}{n}\overset{n}{\underset{k=1}{\sum }}\frac{1}{y_{i}^{2}}\right). \end{array}$$
 Nominal is better:
  (8) $$\begin{array}{c} S/N=-10\log\left(\frac{1}{n}\overset{n}{\underset{k=1}{\sum }}\frac{\mu^{2}}{\sigma^{2}}\right), \end{array}$$

where *μ* = (1/*n*)∑_*k*=1_^*n*^ *y*_*i*_;  *σ* ^2^ = (1/(*n* − 1))∑_*k*=1_^*n*^(*y*_*i*_ − *μ*)^2^.

And *y*_*i*_ represents response variables and “*n*” donates the number of experiments.

One of the distinct features of Taguchi method is that it determines optimum value in the form of *S*/*N* ratio. The predicted *S*/*N* ratio at optimal process conditions can be computed by the following mathematical equation [32]:

(9) $$\begin{array}{c} S/N_{\text{predicted}}=\overset{-}{S}/N+\sum \left(S/N_{j}-\overset{-}{S}/N\right), \end{array}$$

where $\overset{-}{S}/N$ shows the mean of all *S*/*N* ratios, *S*/*N*_*j*_ is the *S*/*N* ratio at the optimal level for each parameters, and *n* is the number of process parameters that significantly affect the process.

## 3. Case Study

In this study, Fenton oxidation of synthetic dye acid blue 113 has been taken as a case study for comparing two optimization techniques, that is, CCD and Taguchi method.

### 3.1. Experimental Details

For performing Fenton oxidation, all reagents used were of analytical grade and procured from Merck Sdn Bhd, Malaysia. Synthetic dye acid blue 113 was purchased from Sigma-Aldrich (M) Sdn Bhd, Malaysia.

For performing Fenton oxidation, stock solutions of 33.3 g/L of H_2_O_2_ and 100 g/L of FeSO_4_ *·*7H_2_O were prepared. All solutions were prepared by using distilled water. Fenton oxidation experiments were performed in batch laboratory scale Erlenmeyer flask (500 mL capacity) equipped with a magnetic stirrer. For each test, 150 mL of acid blue 113 dye solution was placed into flask and pH was adjusted by using 0.5 M H_2_SO_4_ or 1 M NaOH solution according to the designed experiment. Desired amounts of FeSO_4_ *·*7H_2_O and H_2_O_2_ solutions were added to the dye solution, respectively, while stirring at 150 rpm. The start time was recorded when H_2_O_2_ solution was added. After 90 minutes, pH of the solution was measured and sufficient amount of 1 M NaOH solution was added to increase pH above 11 to stop the reaction. This is because in alkaline conditions Fe^+2^ precipitates out as Fe^+3^ and H_2_O_2_ decomposes into oxygen. Then, after 2 hrs of settling time, the supernatant was filtered through 0.45 *μ*m Millipore filter and subjected to various analyses. All experiments were performed at room temperature and atmospheric pressure.

### 3.2. Analysis

pH measurements of the solution were carried out with the aid of pH meter (CyberScan pH 300, Eutectic instruments). Quantitative measurement of the residual H_2_O_2_ in treated wastewater was carried out by using peroxide test strips (Merck). The raw and treated samples were scanned using UV-spectrophotometer (Spectroquant Pharo 300, Merck). And decolorization efficiency of the treated sample was calculated as follows:

(10) $$\begin{array}{c} \text{Decolorization}\left(\%\right)=\left(1-\frac{{\text{Abs}}_{f}}{{\text{Abs}}_{\circ }}\right)\times 100. \end{array}$$

COD measurements were made according to the standard method (APHA, AWWA, and WFE, 1998). For this, COD test cell supplied by Merck was heated in thermoreactor (Spectroquant TR 420) after adding required amount of the sample followed by the subsequent measurement in UV-spectrophotometer according to the standard method. And COD removal efficiency is calculated as

(11) $$\begin{array}{c} \text{COD}\left(\%\right)=\left(1-\frac{{\text{COD}}_{f}}{{\text{COD}}_{\circ }}\right)\times 100. \end{array}$$

## 4. Result and Discussion

### 4.1. Central Composite Design

All experiments for Fenton oxidation were designed according to RSM using central composite design (CCD) with the aid of design expert (Version: 8.0.7.1). According to the literature review, the most important parameters, which affect the efficiency of Fenton process, are pH, initial concentrations of Dye, Fe^+2^, and H_2_O_2_ [4, 33, 34]. Therefore, [Dye]_ini_, pH, H_2_O_2_ : Fe^+2^ (wt/wt), and Dye : Fe^+2^ (wt/wt) ratios were chosen as control variables to be optimized using CCD as given in Table 1.

Total 30 runs with 16 factorial, 8 Axial, and 6 center points were suggested by design expert to optimize the responses, that is, COD and decolorization efficiency. Based on the proposed model, the following quadratic equation was developed to predict the dependent variables (responses) in terms of independent variables and their interactions:

(12) Y=  b0+b1x1+b2x2+b3x3+b4x4+b11x12+b22x22+b33x32+b4x42+b12x1x2+b13x1x3+b14x1x4+b23x2x3+b24x2x4+b34x3x4,

where *Y* is the response variable (COD removal or decolorization efficiency), *b*_0_ is constant, *b*_1_, *b*_2_, *b*_3_, and *b*_4_ are coefficient for linear effects, *b*_11_, *b*_22_, *b*_33_, and *b*_44_ are quadratic coefficient, and *b*_12_, *b*_13_, *b*_14_, *b*_23_, *b*_24_, and *b*_34_ are interaction coefficients [36], respectively.

### 4.2. Model Results for Fenton Oxidation of Acid Blue 113

#### 4.2.1. Model Fitting and Analysis of Variance (ANOVA)

Different concentrations of C.I. acid blue dye 113 were prepared and Fenton oxidation was performed under different conditions according to the experimental runs as suggested by CCD model. Based on experimental results, the following empirical second order polynomial equations were developed showing the interactions between the proposed independent variables to obtain decolorization and COD removal efficiencies:

(13) COD=64.18620−0.20098∗(Dye)+0.70063 ∗(H2O2:Fe+2)+1.75866∗(Dye:Fe+2) ∗0.42339∗(pH)+3.48465E−003 ∗(Dye∗Dye:Fe+2)−1.74043E003 ∗(Dye∗Dye:Fe+2)+0.013948∗(Dye∗pH) +0.031013∗(H2O2:Fe+2)∗(Dye:Fe+2) −0.13479∗(Dye:Fe+2)∗(pH) +3.88643E−004∗(Dye2)−0.066812 ∗(H2O2:(Fe+2)2)−0.021456∗(Dye:(Fe+2)2)Decolorization =85.85276+0.092035∗(Dye)+1.43440  ∗(H2O2:Fe+2)−0.70074∗(Dye:Fe+2)  −0.97745∗pH−1.26375E−003  ∗(Dye∗H2O2:Fe+2)−1.01250E−004  ∗(Dye∗Dye:Fe+2)+0.016293∗(Dye∗pH)  +0.019450∗(H2O2:Fe+2)∗(Dye:Fe+2)  −0.016857∗(H2O2:Fe+2)∗(pH)+0.036054  ∗(Dye:Fe+2)∗(pH)−2.50533E−004  ∗(Dye2)−0.036003∗(H2O2:(Fe+2)2)  −0.50615∗(pH2).

**Figure 4:**
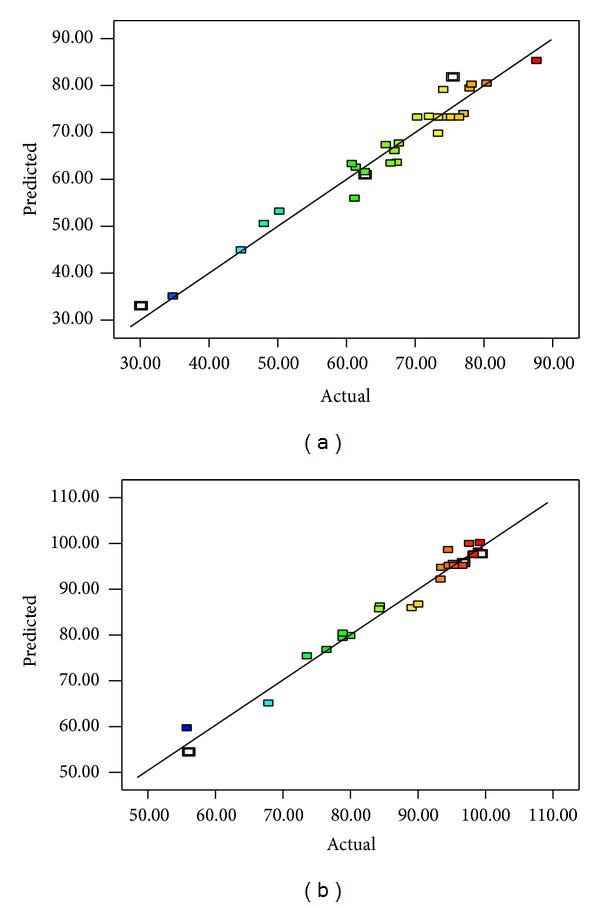
Predicted and actual value for (a) COD and (b) decolorization efficiency.

The objective of this empirical model was to adequately describe the interaction of factors influencing the process efficiency at the concentration ranges investigated. Experimental and predicted values on COD removal and decolorization efficiencies are provided in Table 2. The observed COD removal and decolorization values vary between 30.2–87.7% and 56.1–99.23% which are in good agreement with the predicted values as shown in Figure 4.

Mathematical equation developed after fitting the function to the data may give misleading results and cannot describe the domain of the model adequately [37]. That is why ANOVA is an integral part of the data analysis and is the more reliable way to evaluate the quality of the model fitted [21].  Table 3 shows the analysis of variance for COD and color removal efficiency.

Values of Prob > *F* less than 0.0500 imply that the model terms are significant while values greater than 0.1000 are demonstrated as insignificant for the regression model. In the current study, *F* values 32.41 and 49.73 for COD and color removal denote the model as significant. The goodness of the fit of the model is also checked by *R* ^2^ value. In both cases, the values for this regression coefficient were 0.9789 and 0.9581 which implies that this model is statistically significant and is in reasonable agreement with the adjusted *R* ^2^. Moreover, “Adeq precision” is used to determine the signal to noise (*S*/*N*) ratio to determine the validity of the model. A ratio greater than 4 is recommended. In our case, *S*/*N* values of 25.9 and 22.3 for color and COD removal indicate an adequate signal. They also show that this model can be used to navigate the design space. Another checkpoint for validating the experimental data is the analysis of normal probability plots. The normal probability plot indicates whether the residuals follow a normal distribution, in which case the points will follow a straight line, as it is in the current study (Figures A.1 and A.2 in Supplementary Material available online at http://dx.doi.org/10.1155/2014/869120). Considering the above explained ANOVA results, it can be concluded that this model explained the Fenton reaction and can be employed to navigate the design space in terms of decolorization and COD removal efficiencies.

#### 4.2.2. Response Surface Plotting and Optimization of Operating Parameters

Graphical interpretation of interactions by using three and two dimensional plots of regression model is highly recommended and is used to assess the interactive effects between the process variables and treatment efficiencies of Fenton process [38–40]. The surface response plots of COD and color removal efficiencies of Fenton reaction regarding interaction effects between dye concentration and other selected independent variables are presented in supplementary data in Figures A.3–A.6.

The interaction for assessing decolorization efficiency implies that Dye/Fe^+2^ and H_2_O_2_/Fe^+2^ have proportional effect on color removal efficiency. It can be seen from the plots that there is an increase in decolorization efficiency with increase in control variables and dye concentration. The reason for this increasing trend is that HO^•^ radically produced is reactive enough for the cleavage of the Azo bonds (–N=N–) and at high values of H_2_O_2_/Fe^+2^ and Dye/Fe^+2^, higher concentrations of HO^•^ radical are available to react with the high concentration of dye [33]. In this study, 95% decolorization of acid blue dye was observed within 10 minutes of reaction time under optimum conditions. And over 90% of the color removal was observed for most of the treated samples. However, from data available in Table 2 and contour plots (Figure A.3), it was found that pH exhibits inverse relationship with dye decolorization efficiency. This is because at higher pH values, decomposition of H_2_O_2_ to oxygen and conversion of ferrous ions to ferric hydroxocomplex take place which will in turn make HO^•^ radical unavailable for the reaction to take place [41].

From aforementioned discussion, it is confirmed that Fenton's reagents are active for decolorization. However, efficient degradation of dye is difficult to achieve and it requires optimization of the Fenton oxidation. Unlike decolorization, concentration of Fenton's reagents is not directly linked with COD removal efficiency. In order to make things understandable and comparable, a detailed analysis of the effect of Fenton's reagent on COD removal efficiency is presented in Table 4. Moreover, three- and two-dimensional contour plots obtained by CCD model are also provided in supplementary data (Figures A.4–A.6).

Optimization study of the experimental results was performed by keeping all responses within desired ranges by using response surface methodology. In present studies, dye concentration was targeted to the maximum and other variables were kept in range. Based on the suggested values as given in Table 5, experiment was conducted to validate the optimized results. According to the results obtained approximately, 79.1% of the COD removal was achieved with 98.401% decolorization efficiency. This indicates a good agreement of the experimental and predicted results under optimized conditions. Comparison of the results with previously conducted studies indicates that efficiency of the process is improved in terms of both chemical consumptions and COD removal efficiency. For example, Meriç et al. [42] obtained 71% COD and 99% color removal at optimum conditions of 100 mg/L of FeSO_4_ and 400 mg/L of H_2_O_2_ for 100 mg/L of Reactive Black 5. In another study, 2000 mg/L of H_2_O_2_ and 200 mg/L of FeSO_4_ were used for 88% COD removal of 200 mg/L of Reactive Black 5 [43]. It implies that CCD under RSM is a suitable tool for optimizing Fenton treatment of the recalcitrant wastewater with improved efficiency and less consumption of chemicals.

## 5. Taguchi Method

### 5.1. Experimental Design

Taguchi method was used to determine the optimum conditions for Fenton oxidation and to make comparison with the results obtained with CCD. Minitab 16 was used for the orthogonal experimental design. Same as before, [Dye]_ini_, Dye/Fe^+2^ (wt/wt), H_2_O_2_/Fe^+2^ (wt/wt), and pH were chosen as control factors for optimization through Taguchi orthogonal arrays experimental design. Each factor was varied at three levels (Table 6) and *L*_9_ orthogonal array was selected to determine the optimal conditions with minimum number of experiments. The number of experiments required was reduced to 9 which was considerably lesser than that of CCD. It implies that only 9 experiments with different combination of parameters are required to study Fenton oxidation, which in conventional full factorial design would be 3^4^ = 81 experimental runs.

In second stage, experiments were performed and response values were obtained. For result analysis, Taguchi method follows entirely different steps in contrast to CCD. As followed by previous studies, response values were converted into *S*/*N* ratio which was used to analyze the results [18, 19, 44–46]. For Fenton oxidation, maximum COD and color removal percentages are desired that is why “larger is better” *S*/*N* ratio formula, as given by (3), was used to determine the *S*/*N* value for each response. The experimental results and *S*/*N* ratio for each run are given in Table 7.

In comparison to CCD, ranking of operating parameters in terms of contribution to response values is possible with Taguchi method. It also suggests the use of Taguchi method for screening the input variables during the initial stages of process investigation. In the current study, four parameters were selected and from Table 8, it is clear that for both types of responses pH was the most contributing factor. This observation was also confirmed from the literature that Fenton oxidation shows maximum efficiency at pH 3 and deviation from this value decreases the efficiency of the process [41].

### 5.2. Statistical Analysis

In CCD, analysis of variance (ANOVA) and regression models are used as evaluation criteria for the statistical analysis of the results. However, Taguchi method uses signal to noise ratio (*S*/*N*) as a main approach for the analysis. Nevertheless, ANOVA can be employed for the evaluation of experimental results with a main objective of determining the contribution of each factor to variance of result. It is similar to regression analysis which is used to study and model the relationship between response and one or more independent variables [47].

Table 9 shows ANOVA results for both COD and color removal efficiency of acid blue 113 dye. From the table, it is clear that pH exhibits maximum contribution with percent contribution of 66.2% and 87.62% for both color and COD removal, respectively. It is also in agreement with the values reported in Table 8. Moreover, Sohrabi et al. [19] also reported pH to be the most significant factor (percent contribution: 84.90%) while demonstrating Fenton and photo-Fenton process. This observation can be supported by the fact that, at high pH values, decomposition of H_2_O_2_ to oxygen and formation of hydroxocomplex take place which results in low efficiency of Fenton oxidation even at optimum conditions of Fenton's reagent [41]. Thus, with confidence level of 95%, it has been confirmed that Taguchi method with minimum number of experiments can be used as an alternative to CCD for Fenton oxidation. In order to confirm it, optimized values for the responses were computed to countercheck it with that of CCD.

### 5.3. Confirmation Test and Optimization of Operating Parameters by Taguchi Method

**Figure 5:**
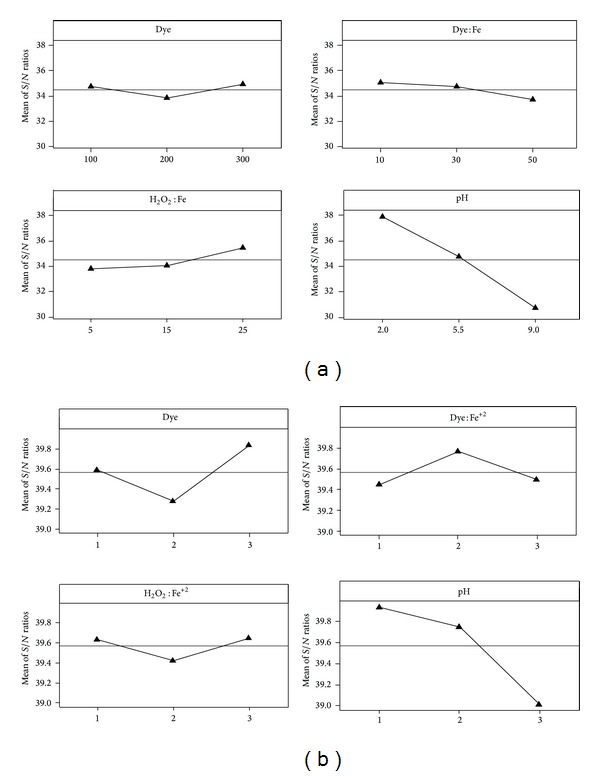
Mean *S*/*N* ratio for (a) COD removal and (b) decolorization.

In Taguchi's method, confirmation test is important to verify the experimental results. However, this method has limitations for multiple response processes like Fenton oxidation. This is because unlike CCD it suggests individual sets of optimized parameters for individual responses. In the present case study, decolorization and COD removal efficiencies were selected as responses for determining the optimized set of the input variables.  Figure 5 shows the best conditions for the Fenton oxidation. The highest *S*/*N* ratios corresponding to the COD and color removal suggest the best levels for each of the parameters. It can be seen from the figure that pH shows the highest *S*/*N* ratio for both COD and color removal efficiencies. Thus, it confirms the maximum contribution of pH to COD and color removal efficiencies.

Figure 5 shows the effect of operating parameters on COD and color removal efficiency in terms of *S*/*N* ratio. The highest *S*/*N* ratios corresponding to the COD and color removal is desirable and suggests the best levels for each of the parameters. In the present example, Dye (*L*_3_), Dye : Fe^+2^ (*L*_1_), H_2_O_2_ : Fe^+2^ (*L*_3_), and pH (*L*_1_) were optimized conditions for COD removal efficiency while Dye (*L*_3_), Dye : Fe^+2^ (*L*_2_), H_2_O_2_ : Fe^+2^ (*L*_3_), and pH (*L*_1_) were the optimized set for decolorization. Optimized values corresponding to these levels are given in Table 10.

Evaluation of operating parameters by using interaction plots indicates that dye concentration of 200 mg/L in different combinations with other parameters shows maximum COD and decolorization efficiency. The interaction plots were produced in Minitab and are provided in supplementary data in Figures A.7 and A.8. From the figures, it can be seen that pH at *L*_1_ shows maximum efficiency for all combinations of operating parameters. However, dye concentration at *L*_2_ in combination with Dye : Fe^+2^ of 30 showed 80% COD removal efficiency. Same results were obtained when H_2_O_2_ : Fe^+2^ ratio was set at 25. Similar trend was observed while studying the interactive effects of operating parameters for determining decolorization efficiency and maximum 99% decolorization efficiency was obtained. Comparison of the findings of the interactive plots of both types of statistical techniques showed that Taguchi method with simple graphical presentation can well explain the interaction of operating parameters. The results obtained were in good agreement with each other. Thus, Taguchi method can be used as a substitute for CCD for assessing the experimental results.

Predicted values of the responses for these optimized values can be computed by using (5). This equation considers only significant parameters. Although only pH is significant parameter according to ANOVA analysis, Dye, Dye : Fe^+2^, and H_2_O_2_ : Fe^+2^ have to be optimized to maximize the COD removal and decolorization efficiency. The predicted *S*/*N* ratios for optimized conditions were computed and obtained values were 37.96 and 39.94 for COD and color removal, respectively. The predicted and actual values obtained as a result of confirmation test are also given in Table 9. The predicted values obtained through Taguchi method were almost close to those obtained through CCD except H_2_O_2_ : Fe^+2^ ratios. The H_2_O_2_ : Fe^+2^ ratio is higher when compared to that obtained with CCD; the main reason for this is that with design expert, it is possible to select the desired range for experimental parameters. With CCD, H_2_O_2_ : Fe^+2^ ratio was selected as the lowest one. However, experimental results obtained under optimized conditions (Taguchi method) are close to predicted values as well as those obtained in CCD. This shows that for optimizing operating parameters for Fenton oxidation, Taguchi method is suitable and economical approach and can be used as a substitute to central composite design.

## 6. Conclusion

This study was planned to compare two optimization techniques such as central composite design and Taguchi orthogonal array and Fenton oxidation with four parameters; that is, [Dye]_ini_, Dye : Fe^+2^, H_2_O_2_ : Fe^+2^, and pH were selected as a case study. From this study, the following points can be drawn as conclusion.

- Taguchi method offered nine experiments for analyzing the Fenton oxidation process while CCD suggested 30 experiments.
- At optimized conditions, Fenton process would be able to achieve 81% and 79% COD removal efficiency and 99% decolorization efficiency for both CCD and Taguchi method, respectively.
- *S*/*N* ratios and ANOVA under Taguchi method showed pH to be the most contributed factor with percent contribution of 87.62% and 66.2% for COD and decolorization efficiency, respectively.
- Interactive study of operating parameters by 3D contour plots produced by CCD also exhibits pH and H_2_O_2_ : Fe^+2^ the most significant factors. However, quantification of contribution is not possible with CCD.

Thus, it can be concluded that Taguchi method is a robust statistical tool for experimental design and process optimization. Data analysis and optimization of operating parameters are possible with fewest numbers of experiments, less computational experience, and graphs obtained which are easy to read and understand. The optimized values obtained for both cases were in good agreement with each other which shows the potential of Taguchi method to be used in chemical engineering applications. Therefore, it can be concluded that it can be used as a substitute for central composite design for Fenton oxidation process.

## Supplementary Material

The supplementary data for supporting the information provided in Table 4, Section 4.1. and interaction of parameters for Taguchi method has been provided in Supplementary Material

## Figures and Tables

**Table 1:** Experimental range and levels of process variables.

Independent numerical variables	Coded	Low actual value	High actual value
Dye (mg/L)	*X*_1_	100	300
H_2_O_2_ : Fe^+2^ (wt/wt)	*X*_2_	5	25
Dye : Fe^+2^ (wt/wt)	*X*_3_	10	50
pH	*X*_4_	2	9

**Table 2:** Experimental design matrix, experimental runs, and predicted values on COD removal and decolorization efficiency.

Independent variables (*X*)					Dependent variables (*Y*; (%))			
					Actual values		Predicted values	
Run	Dye (mg/L)	H_2_O_2_ : Fe^+2^ (wt/wt)	Dye : Fe^+2^ (wt/wt)	pH	COD (%)	Decolorization (%)	COD (%)	Decolorization (%)
1	200	15	30	5.5	75.3	95.4	73.2	95.1
2	200	15	30	5.5	76.4	95.4	73.2	95.1
3	300	25	50	9	62.8	96.8	60.9	95.7
4	200	25	30	5.5	73.4	98.1	69.8	97.5
5	300	5	50	9	34.8	80.0	35.1	79.8
6	200	15	30	5.5	73.4	95.5	73.2	95.1
7	100	5	10	9	66.5	55.8	63.4	59.7
8	100	25	50	2	87.7	99.2	85.3	100.0
9	200	15	30	9	65.8	78.9	67.3	80.3
10	300	15	30	5.5	78.3	94.5	80.2	98.6
11	100	15	30	5.5	77.1	90.1	73.9	86.7
12	100	5	10	2	67.0	93.4	66.1	92.2
13	100	5	50	9	30.2	56.1	32.9	54.4
14	200	15	30	5.5	74.5	95.4	73.2	95.1
15	300	5	10	9	77.9	89.1	79.4	85.9
16	200	5	30	5.5	60.8	84.3	63.3	85.6
17	100	25	10	9	48.0	67.9	50.51	65.1
18	200	15	30	5.5	70.3	94.6	73.20	95.1
19	300	25	10	2	67.4	98.9	63.6	98.3
20	300	5	10	2	61.4	95.2	62.5	95.6
21	100	25	10	2	50.3	97.6	53.1	99.9
22	200	15	10	5.5	67.7	98.2	67.7	97.7
23	300	25	10	9	80.4	84.4	80.5	86.3
24	300	25	50	2	75.5	99.4	81.8	97.7
25	200	15	30	2	74.1	98.3	79.1	97.5
26	100	25	50	9	44.7	73.6	44.9	75.4
27	200	15	50	5.5	62.7	93.5	61.6	94.7
28	200	15	30	5.5	73.4	96.6	73.2	95.1
29	100	5	50	2	72.1	76.5	73.4	76.7
30	300	5	50	2	61.2	78.9	55.9	79.4

**Table 3:** ANOVA results for CCD.

Source	Sum of squares	Degree of freedom	Mean square	*F* value	Prob > *F*
COD removal					
Model	4902.21	12	408.52	32.41	<0.0001 (significant)
Dye	173.73	1	173.73	32.41	0.0017
H_2_O_2_ : Fe^+2^	188.48	1	188.48	13.94	0.0012
Dye : Fe^+2^	168.36	1	168.36	14.95	0.002
pH	620.39	1	620.39	13.36	<0.0001
Color Removal					
Model	4310.88	14	307.92	49.73	<0.0001 (significant)
Dye	635.94	1	635.94	102.70	<0.0001
H_2_O_2_ : Fe^+2^	631.55	1	631.55	102	<0.0001
Dye : Fe^+2^	38.90	1	38.90	6.28	0.0242
pH	1331.97	1	1331.97	215.12	<0.0001

**Table 4:** Interaction of operating parameters for COD removal efficiency.

COD (%)						
Effects	Dye (mg/L)	Dye : Fe^+2^ (wt/wt)	H_2_O_2_ : Fe^+2^ (wt/wt)	pH	COD (%)	Reason
Effect of Dye : Fe^+2^ ratio (Figure A.4)	100	10–23	15	3	65–72.5 (increased)	At low dye concentrations, increase in Dye : Fe^+2^ (wt/wt) results in a decrease in Fe^+2^ concentration and increase in H_2_O_2_ addition (H_2_O_2_ : Fe^+2^) which increases the production of HO^•^ radical for dye degradation
		35–50	15	3	72–65 (decreased)	Scavenging of HO^•^ radical by H_2_O_2_ at low concentrations of Fe^+2^ and high concentrations of H_2_O_2_ [3]
	250–300	10–25	15	3	80 (increased)	Optimum amounts of H_2_O_2_ and Fe^+2^ result in the production of HO^•^ radical adequate enough for maximum dye degradation

Effect of H_2_O_2_ : Fe^+2^ ratio (Figure A.5)	100	30	5–10	3	70–72	
	100–150	30	5	3	73–68 (decreased)	Less availability of HO^•^
	100	30	20–25	3	73–68 (decreased)	Scavenging of HO^•^ radical by excess amount of H_2_O_2_
	300	30	10–25	3	74–80 (increased)	COD removal efficiency increases from 74% to 80% because of the proportionate amount of HO^•^ radical production

pH	100–300	10–50	5–25	3	80	80% COD removal efficiency because of the availability of Fe^+2^ and H_2_O_2_ in aqueous medium (optimum conditions of other variables)

(Figure A.6)	100–300	10–50	5–25	9	60	Decomposition of H_2_O_2_ to H_2_O and O_2_ at pH above 4 [3] Deactivation of ferrous catalyst with the formation of ferric hydroxocomplex [35]

**Table 5:** Optimized operating parameters.

Dye (mg/L)	Dye : Fe^+2^ (wt/wt)	H_2_O_2_ : Fe^+2^ (wt/wt)	pH	Predicted responses		
				COD (%)	Decolorization (%)	Desirability
300	25.92	19.15	3	81.64	99.40	0.972

**Table 6:** Factors and levels of orthogonal array.

Parameters	Level 1	Level 2	Level 3
[Dye]	100	200	300
Dye : Fe^+2^	10	30	50
H_2_O_2_ : Fe^+2^	5	15	25
pH	2	5.5	9

**Table 7:** *L*_9_ orthogonal designs, Levels of four factors, and experimental results obtained.

Dye (mg/L)	Dye : Fe^+2^ (wt/wt)	H_2_O_2_ : Fe^+2^ (wt/wt)	pH	Actual values		*S*/*N* ratio	
				COD (%)	Decolorization (%)	COD	Decolorization
1	1	1	1	74.8603	98.9496	36.53	39.90
1	2	2	2	53.0726	97.9920	34.50	39.82
1	3	3	3	36.3128	89.5582	32.67	39.04
2	1	2	3	32.6996	83.6843	30.97	38.45
2	2	3	1	80.9886	99.1548	37.92	39.92
2	3	1	2	42.9658	93.8547	28.50	39.45
3	1	3	2	69.0217	99.7837	36.78	39.98
3	2	1	3	34.7826	94.8740	30.62	39.54
3	3	2	1	57.8804	99.8905	35.25	39.99

**Table 8:** Response table for signal to noise (*S*/*N*) ratio.

Level	Dye	Dye : Fe^+2^ (wt/wt)	H_2_O_2_ : Fe^+2^ (wt/wt)	pH
COD removal				
1	34.72	35.06	33.87	37.96
2	33.84	34.75	34.12	34.77
3	34.94	33.69	35.52	30.77
Delta	1.10	1.37	1.65	7.19
Rank	4	3	2	1

Decolorization				
1	39.59	39.45	39.63	39.94
2	39.28	39.76	39.42	39.75
3	39.84	39.49	39.65	39.01
Delta	0.56	0.32	0.23	0.93
Rank	2	3	4	1

**Table 9:** ANOVA results.

Factors	Degree of freedom	Sum of squares	Mean Square	*F* ratio	*P* value	Percent contribution (%)
COD (%)						
Dye	2	45	45	0.04	0.961	1.32
Dye : Fe^+2^	2	167	167	0.15	0.860	4.89
H_2_O_2_ : Fe^+2^	2	211	211	0.20	0.826	6.17%
pH	2	2995.3	2995.3	21.23	0.002	87.62
Decolorization (%)						
Dye	2	53.3	26.7	0.83	0.482	21.6
Dye : Fe^+2^	2	18.8	9.4	0.25	0.789	7.61
H_2_O_2_ : Fe^+2^	2	9.6	4.8	0.12	0.888	3.87
pH	2	165.1	82.6	6.07	0.036	66.2

**Table 10:** Optimized values for COD removal and decolorization.

	Dye	Dye : Fe^+2^	H_2_O_2_ : Fe^+2^	pH	Predicted	Actual
COD removal	300	10	25	3	79.06	81.2
Decolorization	300	15	25	3	99.31	99.04

## Abbreviations

AOPs: Advance oxidation process
Fe^+2^: Ferrous iron
Fe^+3^: Ferric iron
H_2_O_2_: Hydrogen peroxide
HO^•^: Hydroxyl radical
OFAT: One factor at time
DOE: Design of experiments
CCD: Central composite design
RSM: Response surface methodology
ANN: Artificial neural network
ANOVA: Analysis of variance
*S/N*: Signal to noise ratio.
