# Supplementary material for: A Comparison of Central Composite Design and Taguchi Method for Optimizing Fenton Process
Source: ScientificWorldJournal. 2014 Aug 27;2014:869120. doi: 10.1155/2014/869120 (PMC4167450; doi:10.1155/2014/869120)
Supplement: Supplementary file 1 — The supplementary data for supporting the information provided in Table 4, Section 4.1. and interaction of parameters for Taguchi method has been provided in Supplementary Material [file 869120.f1.pdf]

SUPPLEMENTARY DATA

Figure A.1 Normal Probability plot of quadratic model fit to the data of response COD(%)

Figrue A.2 Normal Probability plot of quadratic model fit to the data of response Decolorization (%)

Figure A.3 Effect of a) H_2O_2: Fe^{+2} b) Dye: Fe^{+2} c) pH

Figure A.4 Effect of Dye: Fe^{+2} on COD removal (a) Two dimensional contour and (b) Three dimensional surface

Figure A.5 Effect of H_2O_2: Fe^{+2} on COD removal (a) Two dimensional contour and (b) Three dimensional contour

Figure A.6 Effect of pH on COD removal efficiency (a) two dimensional contour and (b) Three dimensional surface

Figure A.7 Interaction of parameters for COD removal

Figure A.8 Interaction of parameters for decolorization

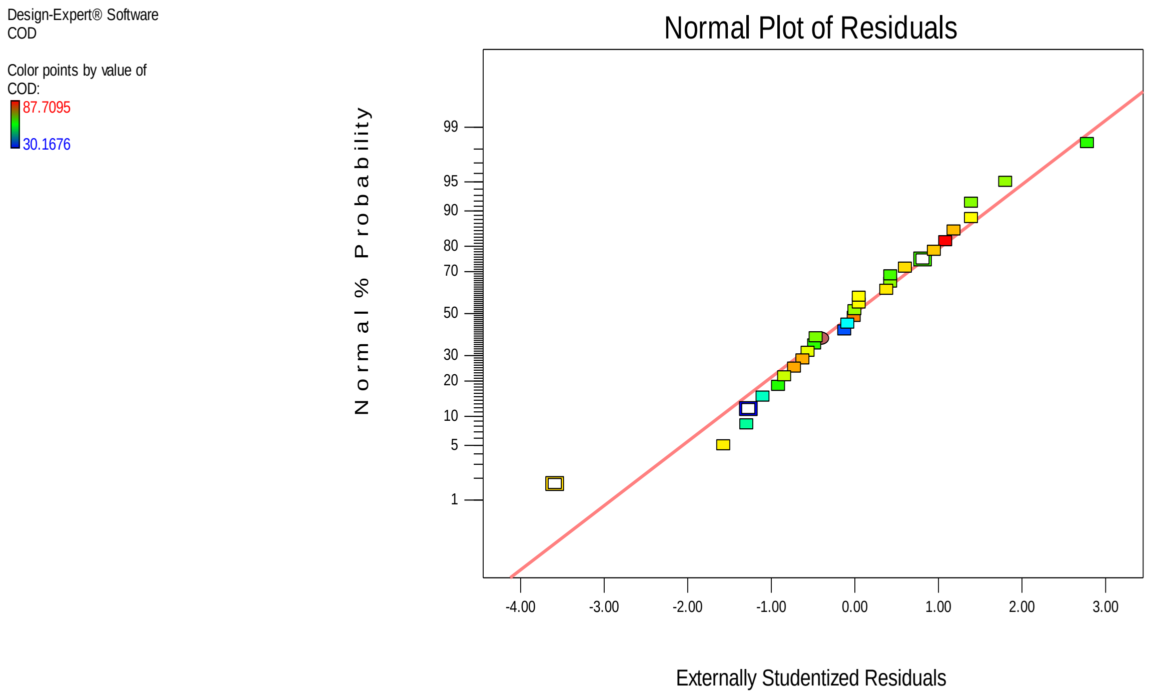

Figure A.1 Normal Probability plot of quadratic model fit to the data of response COD(%)

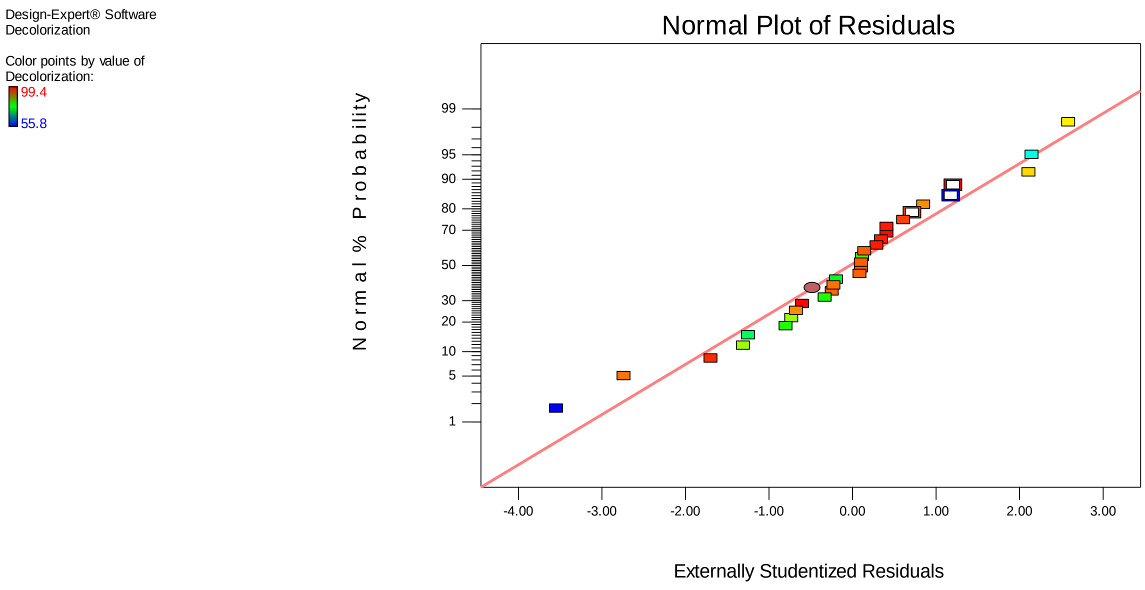

Figrue A.2 Normal Probability plot of quadratic model fit to the data of response Decolorization (%)

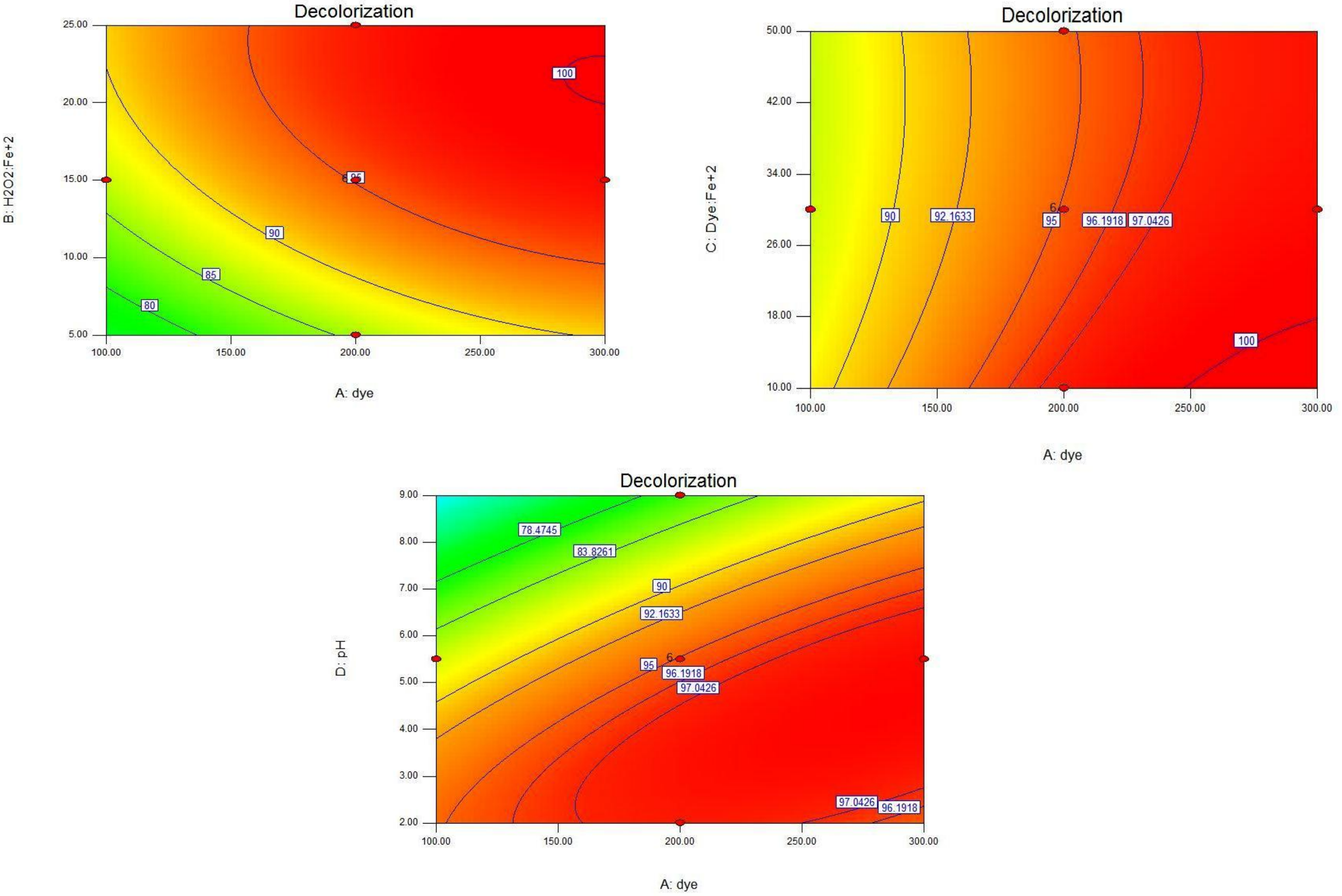

Figure A.3 Effect of a) H_2O_2: Fe^{+2} b) Dye: Fe^{+2} c) pH on decolorization

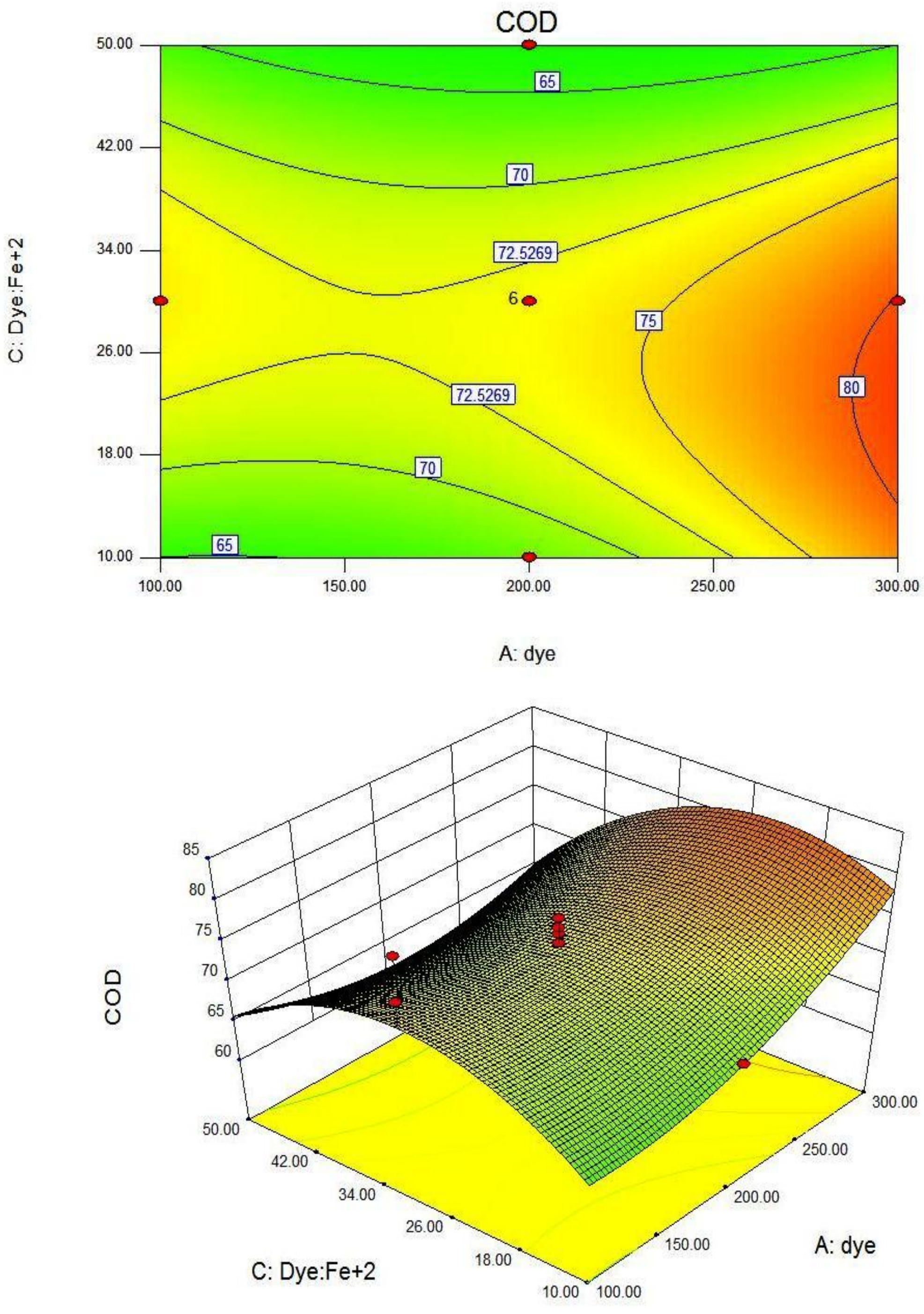

Figure A.4 Effect of Dye: Fe^{+2} on COD removal (a) Two dimensional contour and (b) Three dimensional surface

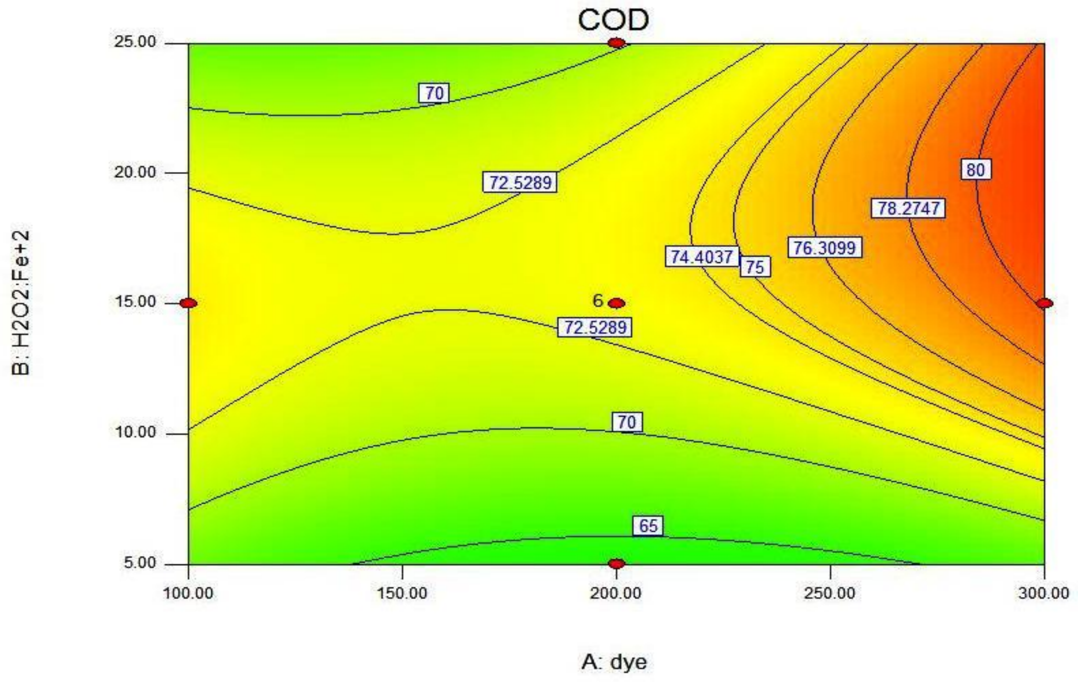

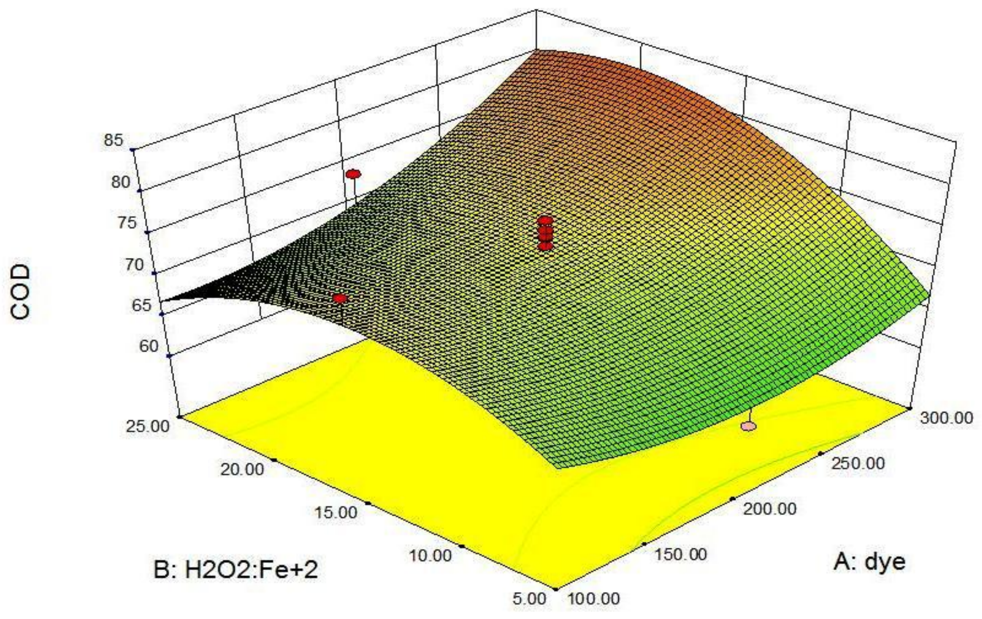

Figure A.5 Effect of H_2O_2: Fe^{+2} on COD removal (a) Two dimensional contour and (b) Three dimensional contour

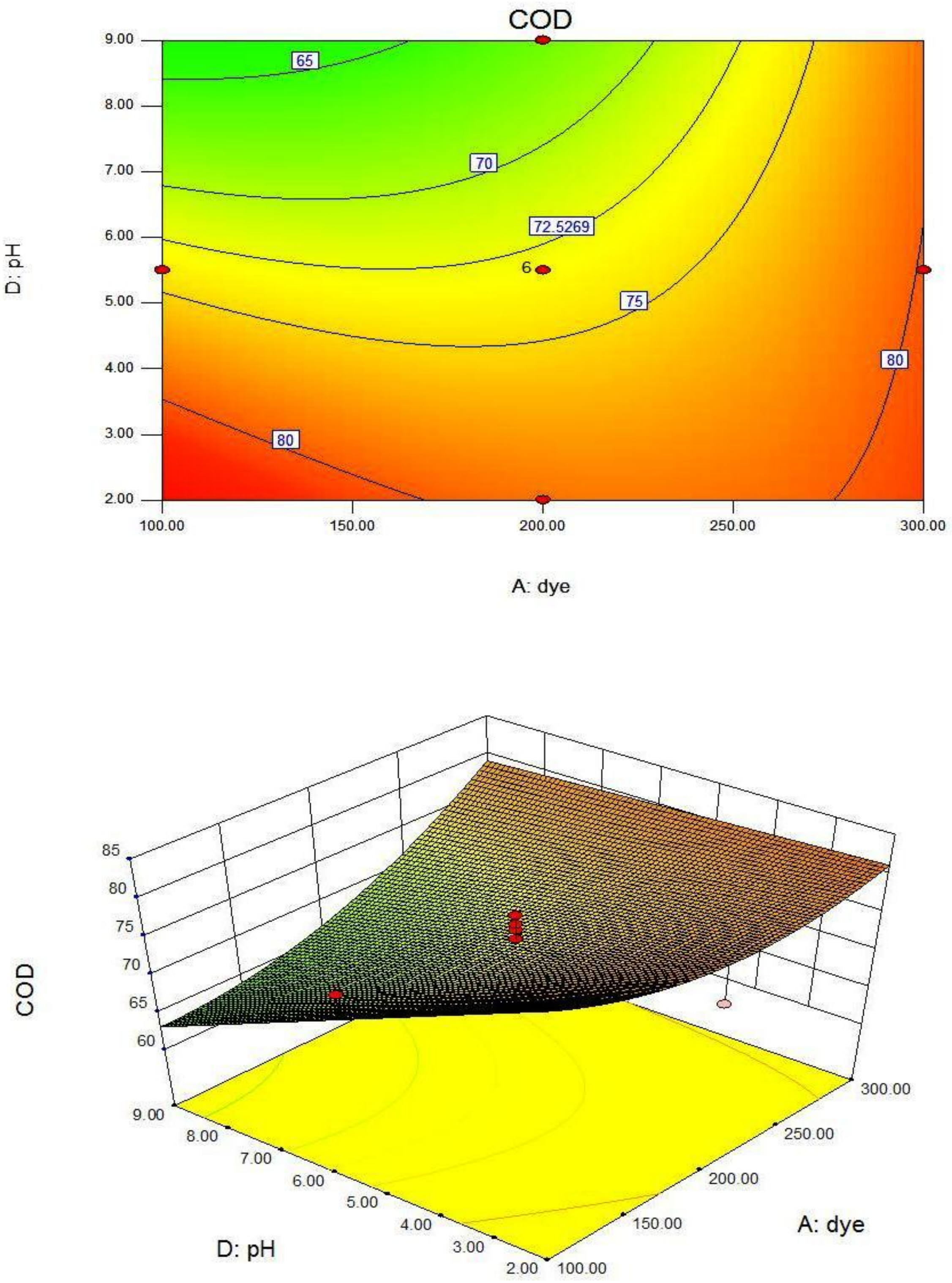

Figure A.6 Effect of pH on COD removal efficiency (a) two dimensional contour and (b) Three dimensional surface

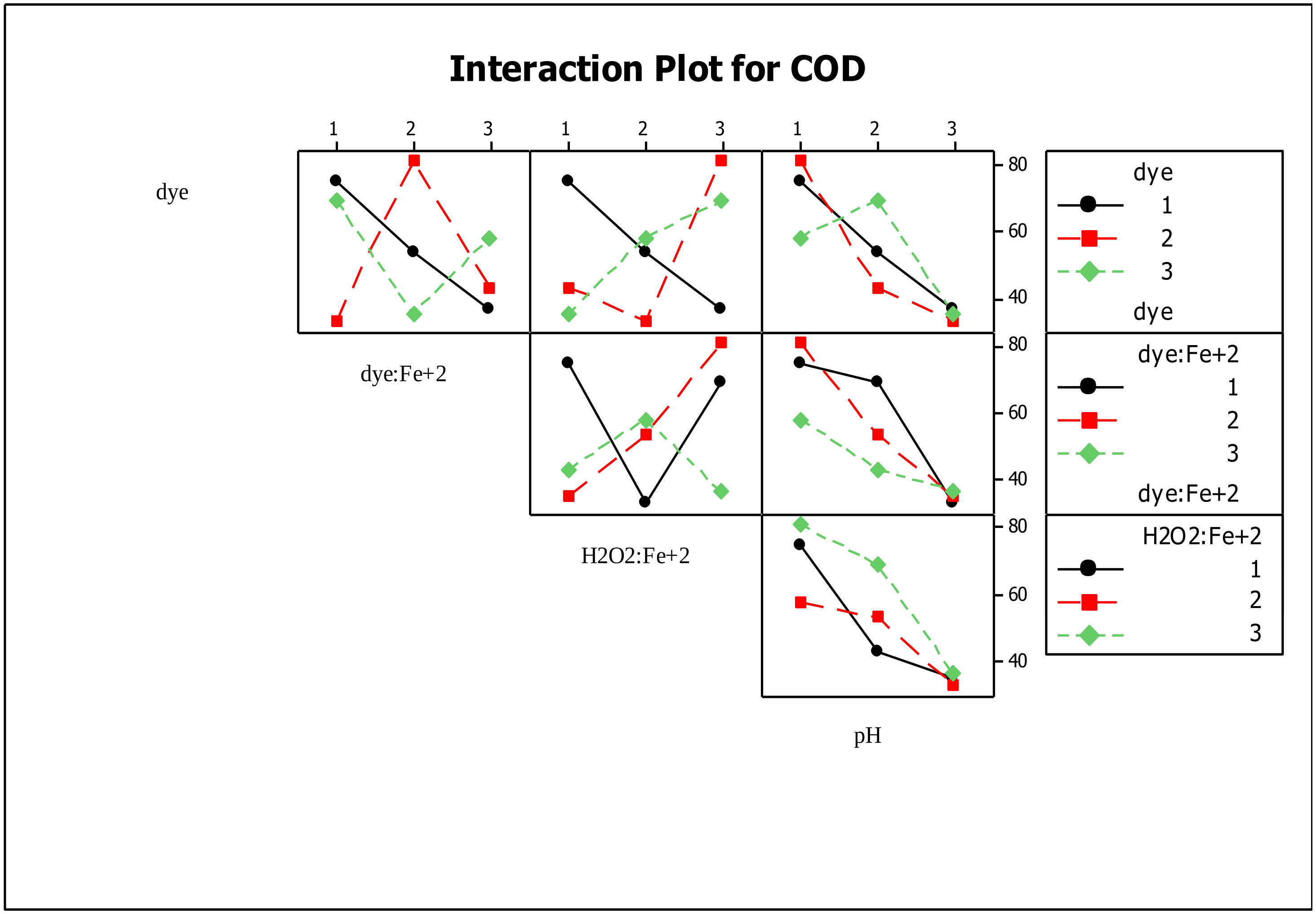

Figure A.7 Interaction of parameters for COD removal

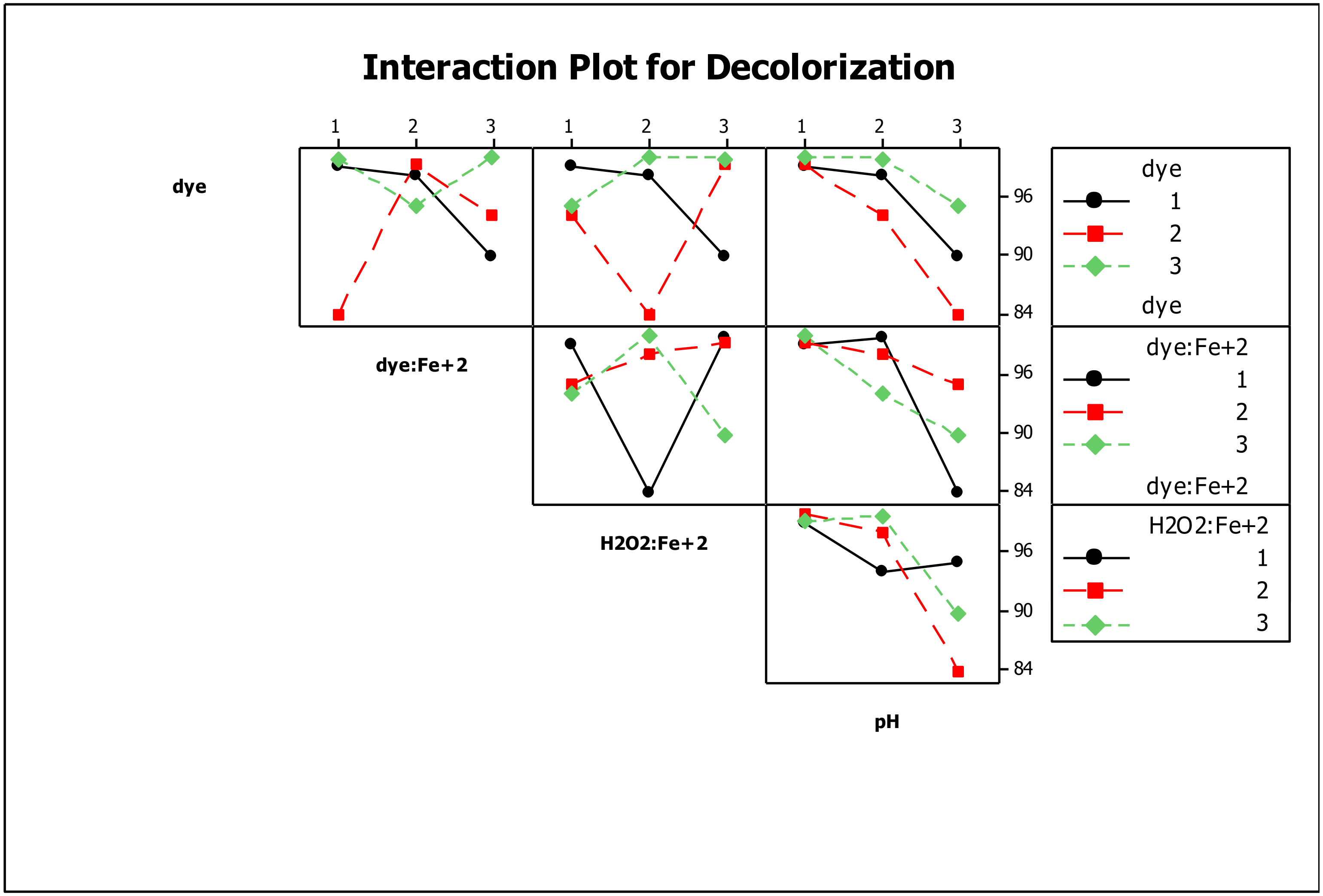

Figure A.8 Interaction of parameters for decolorization
